# An Untargeted LC–MS based approach for identification of altered metabolites in blood plasma of rheumatic heart disease patients

**DOI:** 10.1038/s41598-022-09191-z

**Published:** 2022-03-28

**Authors:** Soumi Das, Yashwant Kumar, Shruti Sharma, Ruma Ray, Sudheer Arava, Sandeep Seth, Aman Agarwal, Gautam Sharma

**Affiliations:** 1 ICMR National Institute of Pathology, Safdarjang Campus, Ansari Nagar, New Delhi, 110029 India; 2 Translational Health Science and Technology Institute, NCR Biotech Science Cluster, 3rd Milestone, Faridabad-Gurugram Expressway, Faridabad, 121001 India; 3 Department of Pathology, All India Institute of Medical Sciences, Ansari Nagar, New Delhi, 110029 India; 4 Department of Cardiology, All India Institute of Medical Sciences, Ansari Nagar, New Delhi, 110029 India; 5 Centre of Integrative Medicine and Research, All India Institute of Medical Sciences, New Delhi, 110029 India

**Keywords:** Biological techniques, Cardiology

## Abstract

Rheumatic heart disease (RHD) is often considered as a disease of developing countries and India is the home of about 40% of RHD patients. Environment seems to play a major role in its causation. Since gene environment interactions can lead to alterations of various metabolic pathways, identification of altered metabolites can help in understanding the various pathways leading to RHD. Blood plasma samples from 51 RHD and 49 healthy controls were collected for the study. Untargeted metabolomics approach was used to identify the metabolites that are altered in RHD patients. Data showed 25 altered metabolites among RHD patients. These altered metabolites were those involved in Purine, Glutamine, Glutamate, Pyrimidine, Arginine, Proline and Linoleic metabolism. Thus, the present study illuminates metabolic alterations among RHD patients which can help in determining the potential therapeutic targets.

## Introduction

Rheumatic heart disease (RHD) is one of the commonest causes of cardiac disease below 25 years of age^1^. Recurrences of Rheumatic fever leads to valvular damage and RHD. The global burden of this disease is estimated to be about 33 million worldwide, causing 2,75,000 deaths annually^1^. Rheumatic fever (RF) and RHD have almost disappeared from high income countries during late twentieth centuries, but are still prevalent in low-income countries. African, South Asian and Pacific Islands are the worst affected regions contributing to almost 84% of the total RHD cases^2^. In the South-East Asian region, India accounts for highest global prevalence of almost 27% (13.17 million) and 1,19,100 deaths approximately^2^. RHD is pathologically heterogenous where gene environment interaction plays a very important role^3^. The pathological mechanism of RHD has not been elucidated in detail till date.

Metabolomics is a powerful technique which has the potential to provide minute details of biological pathways, driven genes or mutations thereby shedding light in understanding the mechanism of disease progression and also discovering diagnostic biomarkers. It is helpful in identifying drug targets for the development of therapeutic agents. Metabolomics approach has helped in detecting biomarkers in diseases like cancer and neurological disorders but to the best of our knowledge there is paucity of metabolomic studies among RHD patients. Metabolomics analysis can be done using untargeted or targeted approach. Untargeted metabolite study is a hypothesis free approach where novel metabolites can be discovered whereas targeted metabolite approach is a hypothesis-based approach where metabolites are known. Thus, metabolomics approach will not only provide insights into the pathogenesis of disease progression but will also help in identifying new therapeutic targets.

In the present study we aim to identify the putative metabolic biomarkers for RHD using high throughput non targeted ultra-high performance liquid chromatography tandem mass spectrometry (UHPLC-MS/MS) which can be useful in early diagnosis and monitoring of RHD, further helping in understanding the disease pathology.

## Results

In this study, a total of 51 RHD patients and 49 age sex matched healthy controls were enrolled. There was no significant difference between the mean age and sex among both the groups. The number of females was higher among RHD patients. The mean body mass index (BMI) was significantly different in both the groups (P = 0.01) (Table 1). The diastolic pressure was significantly high among the RHD patients (P = 0.01) (Table 1). All the patients belonged to New York Heart Association (NYHA) class II and III (Table 1). Only 3.92% were smokers among RHD patients and were not significantly different from healthy controls. Diet pattern was almost similar in both the groups (Table 1).

**Table 1:** Demographic and clinical characteristics of the patients.

	RHD patients (N = 51)	Healthy controls (N = 49)	p value
Age (years)	32.63 ± 8.75	29.93 ± 7.60	0.10
Male n (%)	17 (33.33)	22 (44.89)	0.23
BMI (kg/m^2^)	21.83 ± 3.91	25.71 ± 6.41	**0.01**
Heart rate (beats/min)	78 ± 19.03	79.89 ± 7.79	0.61
Systolic pressure (mmHg)	111.47 ± 14.52	112.17 ± 11.28	0.82
Diastolic pressure (mm Hg)	76.5 ± 11.50	70.28 ± 9.09	**0.01**
EF	57.76 ± 4.86		
MVA (cm^2^)	1.04 ± 0.32		
Severe MS N (%)	28 (54.90)		
**NYHA class**			
NYHA II	34 (66.67)		
NYHA III	17 (33.33)		
Diet veg N (%)	18 (35.29)	24 (48.97)	0.16
Alcohol N (%)	07 (13.72)	15 (30.61)	**0.04**
Smoking N (%)	02 (3.92)	07 (14.28)	0.07

Significant values are in bold.

### Untargeted LC–MS analysis

**Figure 1:**
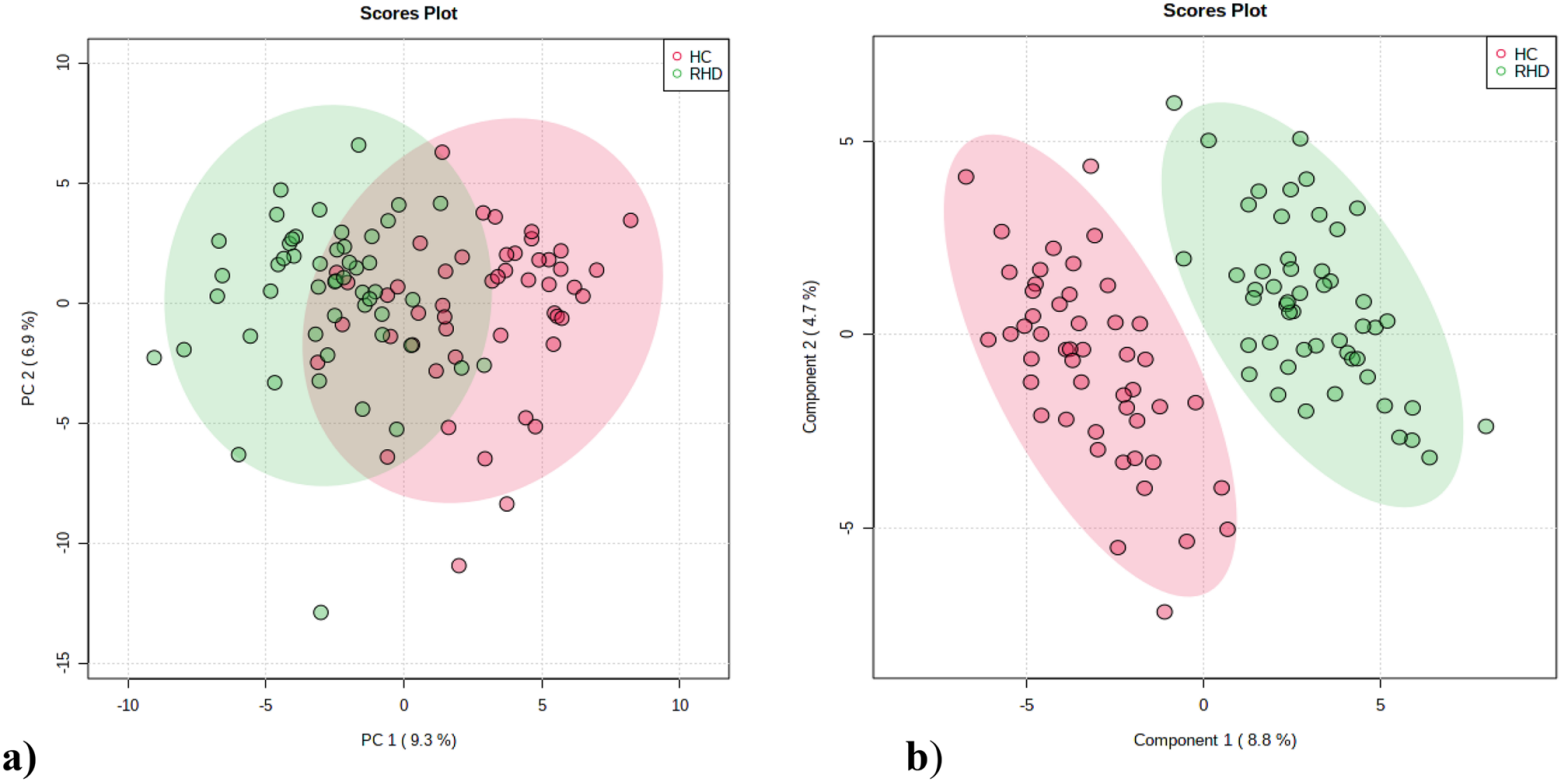
Chemometric analysis of metabolites among RHD and healthy controls. (**a)** Principal Component Analysis (PCA) score plot from RHD and healthy control. The green dots represent RHD patients and the red dots represent healthy controls in the 2D PCA score plots. (**b**) Partial least squares discriminant analysis (PLS-DA) score plot from RHD and healthy control. The two groups are well separated in the PLS-DA score plot, indicating that they had markedly different metabolic characteristics.

351 metabolites were identified in blood plasma through UHPLC-MS/MS analysis (Supplementary Table 1 and Supplementary Table 2). Metabolites with more than 20% missing values were removed from the study rest were replaced by LoDs (1/5 of the minimum positive value of each variable). The peak area data matrix was sum normalized, log transformed and pareto scaled. PCA analysis was performed to understand the aggregation and description of the samples (Fig. 1a.). Whereas PLS-DA score plot helped us to clearly discriminate between the two groups (R^2^ = 0.92 and Q^2^ = 0.76) (Fig. 1b). Cross validation analysis using 100 random permutations were done to prevent overfitting of the PLS-DA model. The R2 and Q2 values of the originally obtained model were better than the 100 randomly permutated models indicating good predictive capacity of the obtained PLS-DA model.

### Identification of significantly altered metabolites

Identification of altered metabolites was based on univariant and multivariant analysis. Consideration of metabolites was based on VIP score > 1.2 from PLS-DA analysis, p < 0.05 selected upon student’s *t* test analysis. 25 metabolites were significantly differentially expressed in the blood plasma samples in RHD patients. Among these 25 metabolites 17 metabolites (*N*-acetylneuraminate, Arachidonic acid, D-Sphingosine, 16(R)-HETE, orotate, inosine, Hypoxanthine, linoleate, Prostaglandin B, d-( +)-Pyroglutamic Acid, l-5-Hydroxytryptophan, Adenosine monophosphate, l-glutamic acid, 5-Methoxysalicylic acid, Prostaglandin A1, d-pantothenic acid, xanthine) were up regulated among RHD patients (Table 2). Remaining 8 metabolites (Caprolactam, trans-4-Hydroxy-l-proline, dihydroxymandelic acid, alpha-Aspartylphenylalanine, 2'-Deoxyuridine, alpha-Lactose, 4-Nitrophenol, 4-Anisic acid) were down regulated (Table 2). Caprolactam, *N*-acetylneuraminate, trans-4-Hydroxy-l-proline, Dihydroxymandelic acid had VIP score > 2 indicating strong difference between RHD patients from the control group (Table 2).

**Table 2:** Plasma metabolites differentiating RHD patients from control group.

	VIP	P value	Log2(FC)	FDR	AUC
Caprolactam	2.3412	3.31E−24	−1.2333	1.16E−21	0.967
*N*-Acetylneuraminate	2.1912	1.27E−05	2.0395	0.000149	0.824
Trans-4-hydroxy-l-proline	2.0438	8.19E−07	−1.6103	2.05E−05	0.779
Dihydroxymandelic acid	2.04	4.05E−06	−2.3744	5.92E−05	0.736
Arachidonic acid	1.8545	2.82E−08	1.4512	1.24E−06	0.805
d-Sphingosine	1.7667	1.99E−05	0.88739	0.000208	0.768
16(R)-HETE	1.7518	2.40E−06	0.71539	4.44E−05	0.733
Orotate	1.7127	0.00309	1.3078	0.013226	0.58
Inosine	1.6591	0.001455	2.44	0.006809	0.719
Hypoxanthine	1.6467	0.005737	1.3465	0.022375	0.747
Linoleate	1.595	0.006022	2.1162	0.023227	0.76
Prostaglandin B1	1.5482	5.03E−06	1.1173	6.61E−05	0.758
d-( +)-pyroglutamic acid	1.4993	3.12E−06	0.8164	5.01E−05	0.773
Alpha-aspartylphenylalanine	1.4817	0.016667	−1.7362	0.047952	0.667
l-5-Hydroxytryptophan	1.4711	3.33E−10	0.70395	3.89E−08	0.876
Adenosine monophosphate	1.4458	0.000781	0.80453	0.004153	0.721
l-Glutamic acid	1.4397	3.27E−06	0.75709	5.01E−05	0.751
5-Methoxysalicylic acid	1.4297	5.09E−06	0.9186	6.61E−05	0.747
Prostaglandin A1	1.4266	6.22E−05	1.1885	0.000555	0.71
2'-Deoxyuridine	1.3609	0.000346	−1.1365	0.002131	0.692
d-Pantothenic acid	1.3421	0.001059	0.83355	0.005236	0.808
Alpha-lactose	1.2985	8.95E−05	−0.73137	0.000748	0.742
Xanthine	1.2797	0.000363	1.3465	0.002158	0.731
4-Nitrophenol	1.2617	9.41E−08	−0.65097	3.30E−06	0.803
4-Anisic acid	1.2602	0.00021	−1.4004	0.001502	0.717

*VIP score* variable of importance score obtained from PLS-DA analysis (VIP > 1.2), *p value* p value of the Wilcoxon signed-rank test, *Log2(FC)* log2 value of fold change, *FC* fold change (FC > 1.5), *FDR* false discovery rate, *AUC* area under the curve calculated from ROC analysis.

Binary logistic regression analysis was also performed to assess the association between altered metabolites and RHD after controlling the effect of BMI and alcohol consumption. Findings of logistic regression analysis are provided in Supplementary table 3.

**Figure 2:**
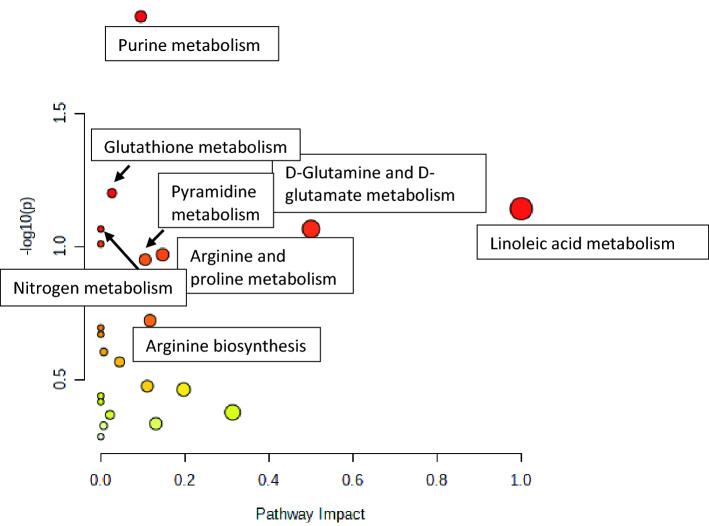
The MetPA analysis based on KEGG Analysis. The darker the red colour of the metabolic pathway, the greater its-log (p) value, indicating a more significant difference.

Statistically significant metabolites were subjected to KEGG pathway analysis (https://www.kegg.jp/kegg/kegg1.html). Purine metabolism pathway was significantly altered (p = 0.01) with more than 2 hits. The results obtained from MetPA analysis is illustrated in Fig. 2.

Impact value more than 0.10 directs that the altered pathway evidently affects RHD patients therefore we consider Purine metabolism, Linoleic acid metabolism, d-Glutamine and D-glutamate metabolism, Arginine and proline metabolism, Pyrimidine metabolism, Arginine biosynthesis, Galactose metabolism, Alanine, aspartate and glutamate metabolism, Arachidonic acid metabolism and Tryptophan metabolism.

## Discussion

Till date there is scarcity of studies to understand the metabolomic changes in blood plasma of RHD patients. Metabolomics is a new approach after genomics and proteomics, which is being used extensively to identify disease biomarkers and biological systems^4^. Metabolomics approach has been used in detecting biomarkers in diseases like cancer, neurological disorders, infectious diseases, inflammation and also in cardiovascular diseases^5^. It is also used to understand the regulation of pathways of various biological processes.

In the present study untargeted LCMS based metabolomics approach has been used to identify potential metabolites for RHD. To the best of our available findings the present study is the first to understand the potential difference between RHD patients and Healthy controls. The main finding of the present study includes identification of 25 significantly altered metabolites, 17 upregulated and 8 down regulated in RHD patients compared to healthy controls.

The 25 significantly altered metabolites were mapped for different pathways. The most important altered pathways were Purine metabolism, Linoleic acid metabolism, D-Glutamine and D-glutamate metabolism, Arginine and proline metabolism, Pyrimidine metabolism, Arginine biosynthesis, Galactose metabolism, Alanine, aspartate and glutamate metabolism, Arachidonic acid metabolism and Tryptophan metabolism (Fig. 2). Notably, purine metabolism comprises increase in inosine, adenosine monophosphate, hypoxanthine and xanthine (Table 2). In ischemic pig myocardium hypoxanthine accumulation has been reported earlier^6^. Xanthine oxidase metabolizes hypoxanthine to xanthine and uric acid. Increased level of hypoxanthine is mainly due to deficiency in hypoxanthine guanine phosphoribosyl transferase (HGPRT). It has been reported in earlier studies that hypoxanthine can lead to endothelial dysfunction by oxidative stress induced apoptosis^7^. Thus, the present study may suggest that hypoxanthine imbalance may lead to RHD.

Other significant altered pathways discovered in RHD patients were d-Glutamine and d-glutamate metabolism and Linoleic acid metabolism (Fig. 2). Glutamate and glutamine are nonessential amino acids that are transformed into each other by glutamine synthase and glutaminase. Framingham heart study reported that the circulating glutamate levels lead to cardiometabolic risk factors whereas circulating level of glutamine and the glutamine:glutamate ratio exhibits opposite association with the cardiometabolic risk factors^8^. Yan Zheng et al., 2016 has reported association of CVD and especially stroke with metabolites in the Glutamate pathway^9^. Since patients of RHD with AF have a high risk of stroke, thus the present study gives evidence for considering glutamate as an early marker for RHD.

Linoleic acid is predominant n-6 polyunsaturated fatty acid (PUFA) which is commonly obtained from vegetable oils and nuts. It has been previously reported that linoleic acid reduces LDL cholesterol thus lowers the risk of chronic heart disease^10^. Therefore, polyunsaturated fatty acid (PUFA) has been recommended for prevention of chronic heart disease. Higher concentration of Linoleic acid shows a proinflammatory and thrombogenic effect^11^ but this result has not been confirmed by randomised controlled trials. Chowdhury et al., 2014 performed meta-analysis and reported no significant association between n-6 polyunsaturated fatty acids (PUFA) and chronic heart disease^12^. Since inflammation and thrombogenic effect are common among RHD patients therefore high consumption of n-6 polyunsaturated fatty acids (PUFA) should be discouraged.

In ischemic rat heart, UTP and CTP degrades quickly compared to ATP^13^. Coronary heart disease patients also show a great disturbance in pyrimidine nucleotides^14^. There are two main pathways for pyrimidine synthesis: de novo* pathway* which use amino acids and CO_2_to synthesize orotate and *Salvage pathways* uses pyrimidine precursors from the diet or from other tissues. The de novo* pathway* is not very prominent in cardiac tissue^15^ but *Salvage pathways* appears to play a significant role in pyrimidine synthesis in heart tissue. The low efficacy of the de novo* pathway* could be due to limited availability of phosphoribosyl pyrophosphate (PRPP). The low availability of PRPP is due to inefficient pentose shunt of carbohydrate catabolism in myocardium. Studies have shown that administration of orotate increases the pyrimidine nucleotide content in heart tissue^16^. Pyrimidine precursor administration can accelerate the reconstitution of glycogen stores^17^. In cardioplegic arrest orotic acid has provided protection to the heart^18^. Orotic acid has also prevented changes in contractility and sarcolemmal glycoproteins in hamsters with muscular dystrophy^19^. Thus, pyrimidine pathway plays an important role in supporting the myocardium^20^. In the present study Orotate was upregulated which may be a protective mechanism in RHD patients.

Arginine and proline metabolism is also observed to be altered in current study. Arginine is one of the most adaptable amino acid which acts as a precursor for protein, nitric oxide, polyamines, urea, glutamate, proline, agmatine and creatinine^21^. Lower arginine availability has been earlier reported to be associated with cardiovascular risk^22^. Proline and hydroxyproline are amino acids which help in maintaining cell structure and functions.

Further, one of the metabolites caprolactam which is a xenobiotic compound was significantly reduced among RHD patients. The direct association of caprolactam with RHD has not been reported previously. However, caprolactam has been reported to be associated with sensory and dermal irritation, dysmenorrhea among humans^23^. Cardiovascular and respiratory effects have been reported in animals with an increase in blood pressure followed by a decrease and an increased respiratory rate^24^. Further studies are required to establish the role of caprolactam in RHD.

To summarize, the present study is the first study to comprehend the complete metabolic alterations among RHD patients. The untargeted metabolomics approach leads to finding of a broad range of metabolites which will aid in understanding the complete view of key metabolic pathway alteration in RHD. The results suggest alteration of several metabolic pathways including purine metabolism, d-Glutamine and d-glutamate metabolism, Pyrimidine metabolism, Arginine and Proline metabolism and Linoleic acid metabolism. Thus, the findings from the present study can act as a tool for validation and identification of possible targets for future therapeutic executives.

## Methods

### Participants and sample collection

5 ml of intravenous blood samples were collected from 51 RHD patients and 49 age sex matched healthy controls. The plasma from the blood samples was separated and stored at −80 °C until analysis. The patients were included in the study after obtaining written informed consent. 12 lead electrocardiogram and two-dimensional echocardiography was done in all the patients. All methods were carried out in accordance with relevant guidelines and regulations. The study has been approved by the Ethics committee of All India Institute of Medical Sciences, New Delhi and National Institute of Pathology, Indian Council of Medical Research, New Delhi.

### Sample preparation

Frozen samples were thawed at room temperature. Metabolites were extracted using chilled methanol in ratio of 1:3 (plasma: methanol) followed by vertexing and centrifugation at 10,000 rpm for 10 min. The supernatant containing metabolites was then collected in a microcentrifuge tube and was lyophilized. The lyophilized samples were reconstituted in 15% methanol and 5 µl was injected for LCMS analysis. Samples were run in randomised way and all the acquisition has been done in single batch.

### Untargeted LCMS metabolomic profiling

LC–MS acquisition was done using orbitrap Fusion (Thermo Fischer) coupled with ultimate 3000 UHPLC system. Ion source used for positive and negative data acquisition was heated electrospray ion source. Resolution of MS was set to 120,000 for MS1 and 3000 for MSMS. Mass range of data acquisition was 60–900 Da. Extracted metabolites were separated on reverse phase column HSS T3 column (Waters)^25^ before infusing to mass spectrometer. Mobile phase A was water with 0.1% formic acid and mobile phase B was methanol with 0.1% formic acid with flow rate of 0.3 mL/min. Total run time was of 14 min with gradient varying from 1% B to 95% B^26^. Quality control (5ul of all samples) run was used after every five samples to monitor the retention time (RT) shift and signal variations. The RT was in minutes. Data were acquired in data dependent mode with intensity threshold an input. Collision energy was 35 ± 15 for MS/MS. Precursor ion selection was from 100 to 1000 Da and the ion isolation width was 1 Da.

### Data processing

Data pre-processing, RT alignment, deconvolution, feature detection, elemental composition prediction and metabolites annotation was done using Progenesis QI software. Metascope plug of Progenesis QI has been used for annotation of the metabolites, the in-house library with accurate mass, fragmentation pattern and RT for database search^27^. The in-house library compounds were purchased from IROA technology that has ~ 600 commands. Same library with chemical class and other information has been published by Phapale et al. 2021^28^ (https://pubs.acs.org/doi/abs/10.1021/acs.jproteome.0c00930). In Progenesis QI there is metascope plugin which can take .msp file (with fragment mass and their corresponding intensities information). Extract algorithm for spectral similarity match could be found at (https://www.nonlinear.com/progenesis/qi/v2.0/faq/fragment-databases.aspx). Identification of metabolites based MSMS spectral similarity was considered only when the fragmentation pattern was > 30 in Progenesis metascope. MSP file of MSMS was downloaded from MS-DIAL spectral database (http://prime.psc.riken.jp/compms/msdial/main.html#MSP) and same has been used for the identification of metabolites using progenesis metascope. Cut-off for RT match was 0.5 min and spectral similarity was more than 30% fragmentation match in Progenesis QI^25^. All features that had coefficient of variation (CV) less than 30% in pool QC samples were rejected^25^. Further, manual verification of each filtered feature has been to done to select the right peaks.

### Multivariate statistical analysis

Statistical analysis in data was done using Metaboanalyst 5.0. The data matrix was sum normalized, log transformed and Pareto scaled. Principal Component Analysis (PCA) was done to understand the clustering pattern. Partial least squares discriminant analysis (PLS-DA) was conducted to clarify groups among clusters. Goodness of the fit and predictive ability of PLS-DA models were evaluated by R^2^ and Q^2^ values respectively^29^.

Comparison of socio-demographic and clinical characteristics across the two study groups:

Continuous variables in Table 1 were represented as mean ± SD and between group comparison was made by two sample t test. Categorical outcomes were reported as frequency (percentages) and compared with Chi squared test. Stata ver. 14.2 was used to perform the analysis.

### Multivariate logistic regression analysis

Binary logistic regression analysis was performed to assess the association between altered metabolites and RHD after controlling the effect of BMI and alcohol consumption. In logistic regression analysis, RHD and healthy controls were assumed as dependent variables. Metabolite peak area, alcohol consumption and BMI were assumed as independent variables. BMI was categorized as normal (BMI ≤ 22.9), overweight (BMI 23–24.9) & obesity (≥ 25 kg/m^2^)^30^ respectively. Stata ver. 14.2 was used to perform logistic regression analysis.

### Significant metabolites selection

Variables with VIP (variable importance of projection) score greater than 1.2 were considered for discrimination. Student’s t-test (P < 0.05) were adjusted for multiple hypothesis testing using FDR correction. Metabolites with fold change threshold of 1.5 and above were considered in the study. Metabolites passing fold change, VIP, P value and FDR criteria were considered for the study. AUC was calculated from ROC analysis. All the univariate analysis was performed with Metaboanalyst 5.0.

### Pathway analysis

Pathway analysis was done using MetPA in Metaboanalyst 5.0. Information from Kyoto encyclopaedia of genes and genomes (KEGG) and human metabolome database (HMDB) for metabolic pathway analysis was used.

## Supplementary Information

Supplementary Table 1.

Supplementary Table 2.

Supplementary Table 3.

## Data Availability

The datasets generated during and/or analysed during the current study are available from the corresponding author on reasonable request.
